# Association Between Recycled Manure Solids Bedding and Subclinical Mastitis Incidence: A Canadian Cohort Study

**DOI:** 10.3389/fvets.2022.859858

**Published:** 2022-04-22

**Authors:** Annie Fréchette, Gilles Fecteau, Caroline Côté, Simon Dufour

**Affiliations:** ^1^Regroupement FRQ-NT Op+Lait, Saint-Hyacinthe, QC, Canada; ^2^Mastitis Network, Saint-Hyacinthe, QC, Canada; ^3^Department of Pathology and Microbiology, Faculty of Veterinary Medicine, Université de Montréal, Montreal, QC, Canada; ^4^Department of Clinical Sciences, Faculty of Veterinary Medicine, Université de Montréal, Montreal, QC, Canada; ^5^Research and Development Institute for the Agri-Environment (IRDA), Québec City, QC, Canada

**Keywords:** bedding, subclinical mastitis, somatic cell count, recycled manure solids (RMS), dairy cows

## Abstract

Recycled manure solids (RMS) are increasingly used as bedding for dairy cows. However, potential impact of RMS bedding on animal health is not well described. The objective of this study was to evaluate subclinical mastitis incidence in cows housed on RMS bedding. Twenty RMS farms and a comparative group of 60 straw-bedded farms were enrolled in a 1-year longitudinal study (2018–2019). Data from 11,031 dairy cows were collected. Variations of individual somatic cell count were evaluated using three different methods. First, we compared the cow's mean lactation linear score between cows housed on the two bedding types. Then, we compared across bedding types the risk for a given cow of having a milk test with a linear score ≥ 4. Finally, we evaluated the dynamics of somatic cell count using pairs of tests within a cow. More specifically, we considered that only pairs of DHI tests where the first test yielded a linear score < 4 were at risk of an incident subclinical mastitis event. Then, we defined a newly acquired subclinical mastitis when the second test was ≥ 4. All models were adjusted for putative confounders. We could not highlight a significant association between bedding type and cow's mean lactational linear score (least square mean of 2.47 in cows from RMS farms vs. 2.37 in straw farms; 95%CI for linear score's difference: −0.20, 0.40). Furthermore, we could not find an association between bedding type and the risk of a high linear score (≥ 4). For the latter, cows housed on RMS had 0.93 times the risk of having a high linear score than straw-bedded cows (%95 CI: 0.68, 1.28). Moreover, cows on recycled manure solids farms had 0.73 time the risk of acquiring subclinical mastitis when compared to straw-bedded farms. Again, this risk was not statistically significant (%95 CI: 0.54, 1.00). In our study, RMS bedding was not associated with subclinical mastitis, as measured by somatic cell count, when compared to cows housed on a more conventional bedding, straw bedding.

## Introduction

Dairy producers have a growing interest for using recycled manure solids (**RMS**) as bedding. Although RMS is used in several countries, scientific data on the impact of this product on cow's health are scarce. Due to the nature of RMS, there are concerns about the pathogen load in this bedding and the resulting risks for cow's and farm workers' health. Pathogens such as *Salmonella* spp., *Listeria monocytogenes* and *Cryptosporidium* spp. have been isolated from fresh unused RMS bedding (1, 2). Furthermore, *Mycobacterium avium* ssp. *paratuberculosis*, the pathogen responsible of Johne's disease, was also found in unused RMS bedding samples (3).

It has been demonstrated that using RMS bedding influences bulk tank milk microbiota (4). Moreover, bulk tank milk bacterial counts of some bacterial species such as *Streptococcus* spp. and *S*t*reptococcus*-like organisms were higher in farms using RMS compared to other organic beddings (5). It is not clear, however, whether RMS bedding affects bulk milk through a change in mammary gland microbiota, or later by contamination during milk harvesting. Thus, it is still debatable as whether RMS bedding can be used on dairies, without negatively affecting udder health. Some studies have not found an increased risk of total (i.e., all causes together) clinical mastitis (**CM**) cases when RMS bedding is used (6, 7). Others have estimated a higher monthly incidence of CM (4.7%) in RMS-bedded cows compared to cows housed on reclaimed sand (2.1%) (5). Few studies have investigated the effect of RMS on pathogen-specific CM risk, but, in one study, the risk of CM due to *Klebsiella pneumoniae*, specifically, was seven times higher in cows housed on RMS compared to cows housed on straw (7).

Beyond CM, another important measure of udder health is subclinical mastitis (**SCM**). Subclinical mastitis is defined as presence of inflammation in the mammary gland, without observable signs of disease (8). It is often measured using somatic cells count (**SCC**) or linear cells score (**LS**) (9).The latter involves a logarithmic transformation of SCC, and is used because of the very skewed distribution of SCC in most herds (10). In an experimental study, Rowbotham and Ruegg found no association between use of RMS bedding and incidence of SCM (defined as a monthly milk sample with SCC > 200,000 cells/ml) in primiparous cows (6). In an observational study conducted by the Cornell Waste Management Institute (3) in a 1,600 cows herd, the type of bedding used (RMS vs. sand) did not affect the animal's SCC. In a literature review published in 2015, Leach et al. concluded that there was no consistent impact of RMS usage on SCC. On the other hand, use of RMS bedding was recently associated with poorer udder health measures such as the average test day LS, the proportion of cows on test day with a LS ≥ 4.0, and the proportion of cows with a chronic infection when compared to herds using other types of bedding (5).

Objectives of the current study were to contribute to the knowledge on the associations between RMS bedding and SCM by investigating its association with the cows' lactation mean linear score, the risk of SCM on a given test day, and the risk of SCM acquisition, when compared to cows housed on straw bedding. We hypothesized that, given that bedding type would mainly influence intramammary infections of environmental origin, and given that these latter infections have lower impact on the SCC than contagious pathogens, there would be small or no associations between bedding type and SCC-based measures of inflammation. This paper is part of a larger project where we have studied parasitic load and survival in RMS bedding (1), RMS bedding bacteriological content and microbiota (2), the effect of RMS on pathogen-specific CM incidence (7), and the effect of RMS bedding on bulk milk quality (4).

## Materials and Methods

This project was approved by the Animal Care and Use Committee of the Faculty of Veterinary Medicine (Université de Montréal; protocol 17-Rech-1886). We conducted an observational cohort study on commercial dairy farms. This paper was elaborated in agreement with the STROBE-Vet statement (11).

### Herd Recruitment

We aimed at recruiting 90 herds, of which ≥ 20 would be using RMS bedding for lactating cows, and the remainder would be using straw bedding (as a comparative group). This number and ratio were determined by *a priori* power estimations which were conducted for the various outcomes studied [bedding microbiota, (2); parasitic load, (1); clinical mastitis incidence, (7); bulk tank milk quality, (4); subclinical mastitis, hygiene, and comfort]. The number of farms to recruit was determined mainly by the clinical mastitis outcome, which required the largest sample size [see Frechette et al. (7) for details]. Herds using RMS bedding were first identified by contacting equipment dealers, veterinarians, and social media. Straw-bedded herds were invited to participate to the study by the local dairy herd improvement (DHI) company (Lactanet, Ste-Anne-de-Bellevue, QC, Canada). To be included in the study, all farms needed to be within 250 km of the research facilities (Saint-Hyacinthe, QC, Canada) and to have used the same type of bedding for lactating cows for at least 6 months at the time of the visit. Straw-bedded farms needed to record production data using DHI to facilitate the data collection. However, this was not requested for RMS farms since we wanted to recruit as many RMS farms as possible.

All eligible farms were contacted by telephone between July and December 2017 to verify their eligibility and willingness to participate to the project. Descriptive data (such as farm size and type of equipment used for producing bedding) were gathered from RMS herds that did not meet the inclusion criteria.

### Data Collection

Farmers agreed to share their monthly DHI data for the follow-up period (1 year following the visit of the farm). In the straw group, we selected herds that also used a software to record their individual cow's health data. In the RMS group, some farmers were not participating in regular DHI program. When milking system recordings of SCC were available, these data were obtained. Producers with no DHI and no milking system-based SCC were excluded from the subsequent analyses. For SCC data obtained from the milking system, the data were converted as described by Deng et al. (12) to generate a dataset that was uniform between herds participating or not in DHI. Briefly, a daily mean LS based on a 24 h period was computed and, subsequently, a monthly average LS was generated.

### Outcomes Studied

We analyzed SCM using three different outcomes. First, we computed the cow's lactational mean LS. Secondly, we computed, for each cow, the number of DHI tests with a LS ≥ 4 among the number of available DHI tests. Finally, we computed, for each cow, the number of incidents SCM events among all the periods at risk for that cow. For this third outcome, DHI tests were considered in pairs. During the period between two DHI tests, a cow was defined as being at risk of developing a SCM if it had a LS < 4 on the first test of the pair. Among cows at risk of acquiring a SCM, an incident SCM was deemed to have occurred if a LS ≥ 4 was observed on the second test. If one of the tests had a missing data, the correspondent test pair was discarded from the analyses. For this third outcome, we were, therefore, able to calculate, for each animal, the sum of SCM events during the study period and the total number of days at risk, as described by Dufour et al. (13).

### Statistical Analysis

To describe the effect of bedding type (RMS vs. straw) on the cow's lactational mean LS, we used a linear mixed regression model. In this model, the outcome was the lactational mean LS and the main predictor was the bedding type. A cow and a herd random intercepts were used to capture the variation due to clustering of lactations by cows and of cows by herd, respectively. A number of putative confounders previously identified using directed acyclic graphs were included in the models to control for confounding: housing type (free stall vs. tie-stall), bedding thickness (<10 vs. ≥ 10 cm of depth), time since the last renovation of the stalls (in years), and herd size (number of milking cows at the time of the visit). For a covariate to be considered as a putative confounder, and thus for inclusion in our models, we applied the following criteria (14): (1) the covariate is a determinant of the exposure in the source population; (2) the covariate is a cause or a surrogate for a cause of the disease; and (3) the covariate is not caused by the exposure nor the disease.

In our second model, the outcome was the number of tests with a LS ≥ 4.0 for a given cow during the study period. To model the effect of bedding on this outcome, we used a generalized linear mixed model with a negative binomial distribution, a log function, and the logarithm of the number of milk tests available for this cow as an offset. Again, the main predictor was the bedding type and the same putative confounders described in the first model were included. Finally, a herd random intercept was included to consider the clustering of cows by herds. Thus, using this model one could compute the incidence rate ratio of having SCM in RMS farms as compared to straw-bedded farms simply by exponentiating the bedding variable coefficient.

In our third and last model, the outcome was the number of times a cow acquired a SCM during the study period. To estimate the bedding effect on that variable, we used a generalized linear mixed model with a negative binomial distribution, a log function, and the logarithm of days at risk for that cow as an offset. Putative confounders and a herd random intercept were, again, included in the model. The incidence rate ratio of having an incident SCM in cows bedded with RMS *vs*. straw could then be computed by exponentiating the bedding variable coefficient.

For each modeling approach, the assumption of linearity between the quantitative predictors (time since the last renovations of the stall and herd size) and the different outcomes was verified by adding polynomial terms (square and cubic terms) after centering the predictor. Polynomial terms were kept in the model whenever they were significant. The significance level was set at *p* < 0.05. If overdispersion was present in the data, defined as a Pearson chi-square > 1.2, then robust variance was used. Statistical analyses were performed with MLwin 3.05 (U of Bristol) for the first model and SAS 9.4 (SAS Institute Inc., Cary, NC) for the second and third model. Finally, normality and homoscedasticity of residuals were evaluated at all levels of the hierarchy (lactation, cow and herd level; for the MlWin analyses), or using marginal residuals (for SAS analyses).The data, the MlWin model and the SAS scripts are publicly available on https://doi.org/10.5683/SP3/GRPK8W.

## Results

### Herds Description

Herds recruited were part of a larger study on RMS bedding and were described elsewhere (1, 2, 4, 7). Briefly, we contacted 49 RMS farms and 139 straw farms to verify their willingness to participate and to evaluate whether they met the inclusion criteria. In the RMS group we excluded 11 farms that where outside the geographic location, six that could not be joined after multiples attempts, four that had changed their bedding to a conventional bedding, and one that was misidentified as using RMS bedding. In total, 27 RMS farms and 61 straw farms were initially enrolled in the study. All had Holstein cows with the exception of one Ayrshire herd (straw bedding), and one Brown Swiss herd (straw bedding).

**Figure 1:**
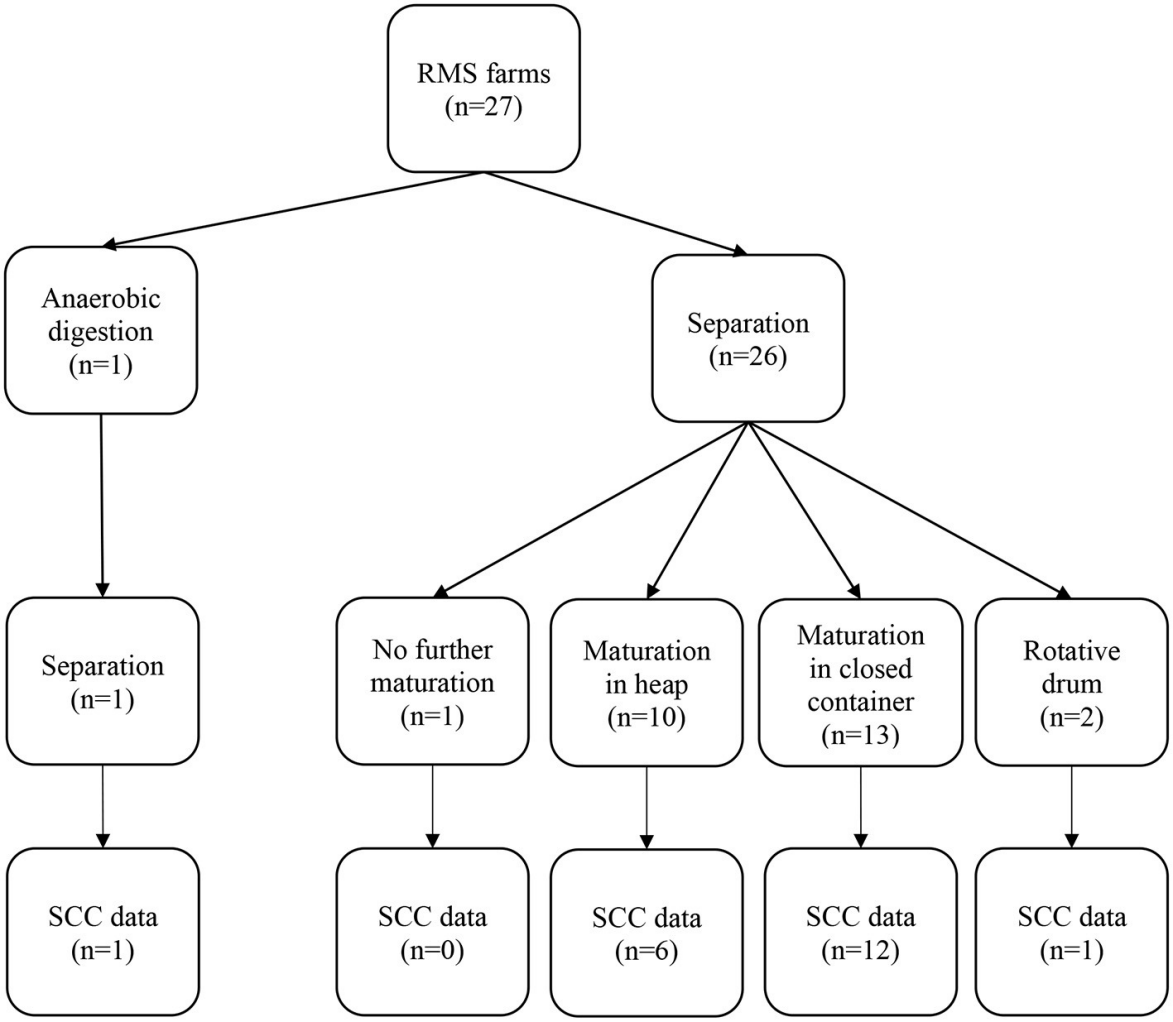
Description of the farms' recycled manure solids (RMS) bedding production procedures and of somatic cell count (SCC) data availability.

As can be seen in Figure 1, RMS farms produced their bedding with various methods. One farm used an anaerobic digester followed by a separation process and one farm used the bedding immediately after the separation process. The other 25 RMS farms let the RMS mature for various amount of time following the separation process (median: 2.5 days; range: 0.4–9 days). Ten of them let it rest in a single heap, 13 in a closed container, and two used a rotative drum.

The earliest initial farm visit was conducted on January 15^th^ 2018, and the latest was conducted on July 10^th^, 2018. DHI data were collected, on each farm, for 1 year following the farm visit. Seven RMS herds were excluded of the SCC analyses because their automatic milking system (Delaval, Milkomax, GEA or Boumatic) did not record any SCC data. Demographic details about the farms with SCC data are shown in Table 1. Briefly, RMS farms were larger and had renovated the stalls more recently than straw farms. Furthermore, RMS farms were predominantly free stall, and almost half of them were using a deep-bedding (≥10 cm) system. There were six herds using automatic milking system in our group of 20 RMS herds with SCC data. Four herds had a Lely robot (which recorded SCC data) and two herds, even if they had robots (1 DeLaval and 1 GEA), where DHI participants. Three farms did not have a complete year of follow-up due to a change in the type of bedding during the year (1 straw farm) or if they experienced a fire (1 RMS farm and 1 straw farm).

**Table 1:** Description of the 20 recycled manure solids farms and 60 straw farms with SCC data included in the study on subclinical mastitis.

	**RMS bedding Median (range)**	**Straw bedding Median (range)**
Number of milking cows	113 (55–900)	65 (43–229)
Somatic cell count^ 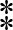 ^	170 (110–304)	187.5 (69–384)
Number of years since the last renovations of the stalls	3 (1–23)	10 (0–70)
Proportion of free stall farms (%)	75	3
Proportion of farms with bedding depth ≥ 10 cm (%)	40	0
Proportion of farms using an automatic milking system (%)	30	3
Proportion of farms participating in DHI (%)	80	100

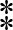
*Mean somatic cell count of the 12 months preceding the farm visit (× 1,000 cells/ml)*.

### Data Collection

Although the participation of straw farms to DHI was a selection criterium, one farm quitted DHI after its recruitment and, therefore, did not collect data during the follow-up period. We have consequently collected DHI data for 60 straw-bedded farms. Sixteen of the RMS farms were also part of the DHI program. Among the 10 additional RMS farms that used an automatic milking system, we were able to obtain SCC data for four farms. We were, therefore, able to collect SCC data for 20 RMS farms. We followed 15,161 lactations from 11,031 cows during the study period (4,618 cows housed on RMS bedding and 6,413 cows housed on straw bedding).

### Model 1: Impact of Bedding Type on the Cow Mean Lactation LS

**Figure 2:**
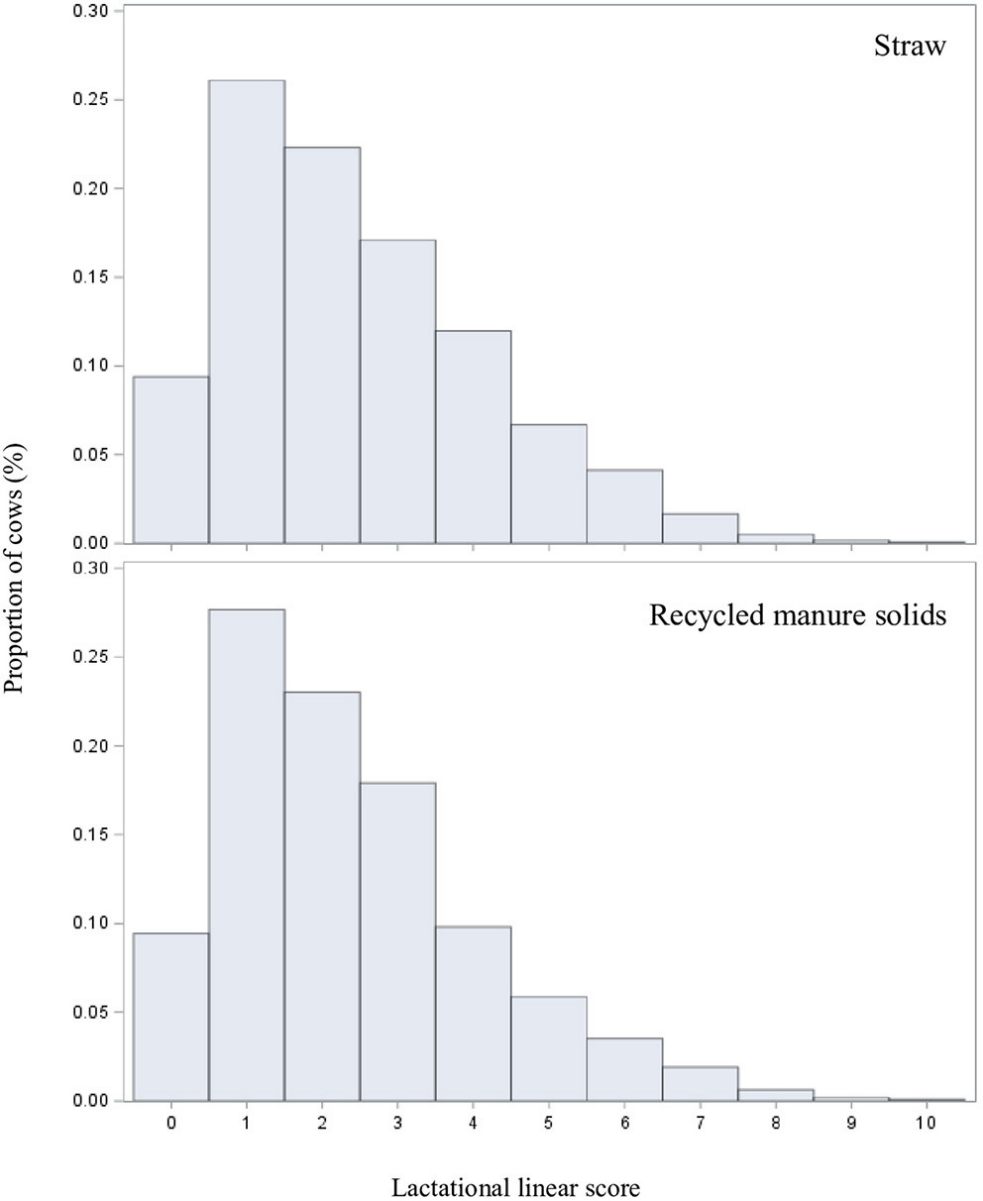
Cow's mean lactational linear score distributions by bedding type for 15,161 lactations of 11,031 cows from 60 straw-bedded farms (top) and 20 recycled manure solids farms (bottom).

For this first model, we obtained data from 15,161 lactations of 11,031 cows which, on average, contributed 1.4 lactation each (range: 1–2). Participating herds had an average of 138 cows (range: 34–1354). The median (interquartile range) number of tests per lactation recorded during the study was 4 (2–7). As we can see in Figure 2, the mean lactational linear score distributions were very similar in the two farm groups. After adjusting for putative confounders, we did not find a significant association between bedding type and mean lactation LS, with a mean LS of 2.47 in RMS farms compared to a mean LS of 2.37 in straw farms (Table 2). Mean lactational LS was estimated to be 0.10 points higher in RMS farms (95%CI: −0.20, 0.40).

**Table 2:** Impact of bedding on the cow's mean lactation linear score estimated using a generalized linear mixed model using the data from 15,161 lactations of 11,031 cows from 20 recycled manure solids (RMS) farms and 60 straw-bedded farms.

	**Coefficient**	**SE**	**CI**	***p***
Intercept^†^	2.37	0.06	2.25, 2.49	
Bedding type
RMS	0.10	0.15	−0.20, 0.40	0.50
Straw	Ref			
Housing type^‡^
Free stall	0.25	0.17	−0.09, 0.59	0.15
Tie stall	Ref			
Bedding depth^ 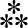 ^
≥10 cm	−0.06	0.19	−0.44, 0.32	0.77
<10 cm	Ref			
Stall age^‡, 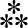 ^	0.09	0.04	0.01, 0.17	0.04
Herd size^ 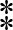 , 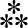 ^	−0.11	0.04	−0.2, −0.02	0.01
Variance
Farm	0.11			
Cow	0.93			
Lactation	1.88			

^†^*Stall age and herd size were centered on 5 years and 100 cows, respectively. The intercept, therefore, represents the cows' mean LS for a cow in a 100 milking cows herd that had renovated its stalls 5 years ago*.

^‡^*Coefficient represent an increase of 10 years*.

^
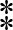
^*Coefficient represent an increase of 100 cows*.

^
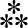
^*Putative confounders*.

### Model 2: Impact of Bedding Type on Risk of a Test With LS ≥ 4 for a Given Cow

**Figure 3:**
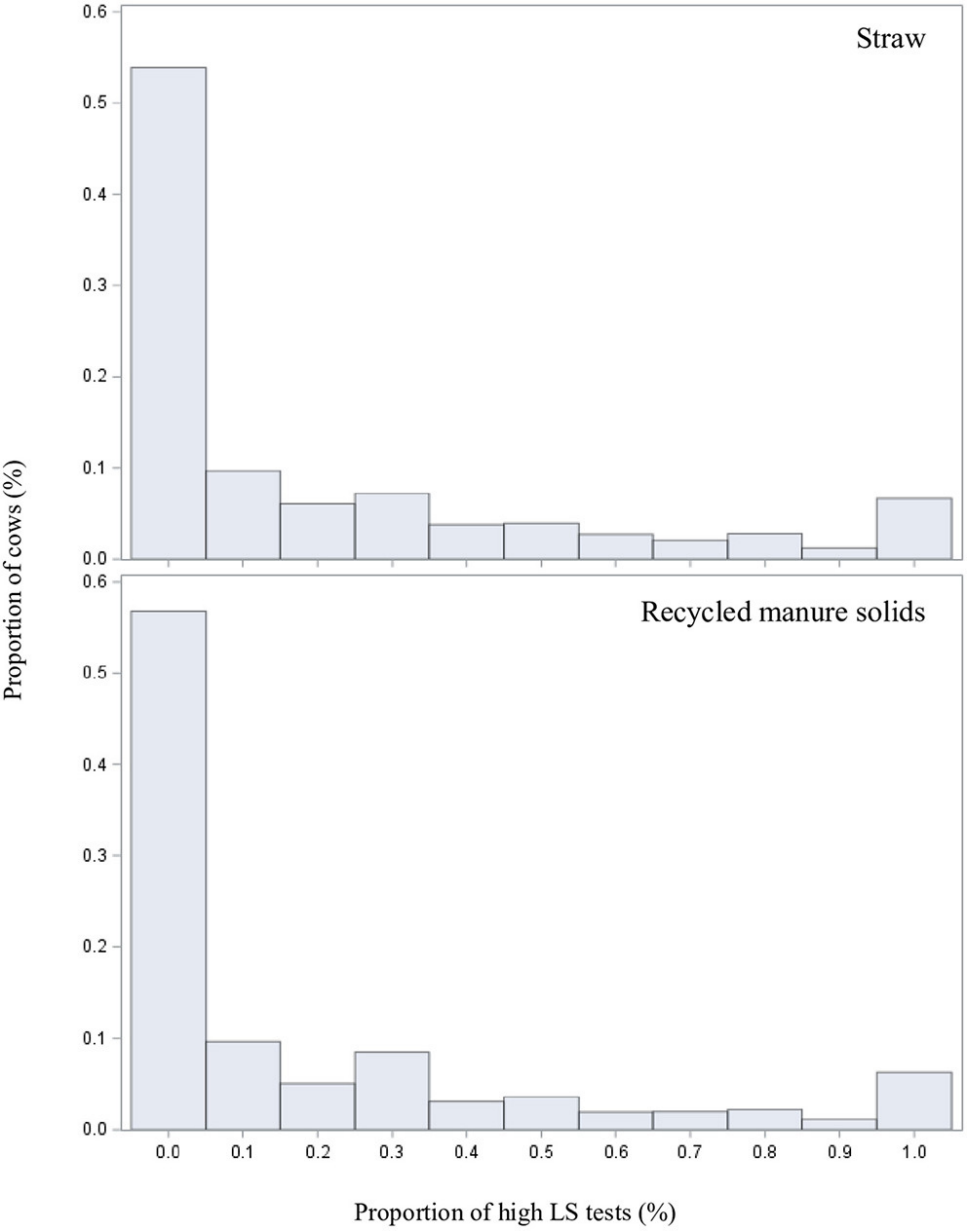
Distributions of the cow's proportion of milk tests with a linear score ≥ 4 by bedding type for 11,031 cows from 60 straw-bedded farms (top) and 20 recycled manure solids farms (bottom).

In this model we used data from 11,031 cows. On average, data from 138 cows per herd (range: 34–1,354) were available. The distribution of the cow's proportion of milk tests with a LS ≥ 4, as function of bedding type, is illustrated in Figure 3. The relationship between herd size and this outcome appeared to be non-linear on the logarithmic scale. Therefore, the square and cubic polynomial herd size terms were retained in the model. After adjusting for confounding, RMS-bedded cows had a 0.93 times (%95 CI: 0.68, 1.28) the risk of having a LS ≥ 4.0 than straw-bedded cows (Table 3).

**Table 3:** Impact of bedding type on the risk of a DHI test with a linear score >4.0 in 11,031 cows from 20 RMS farms and 60 straw-bedded farms and estimated using a generalized linear mixed model.

	**Coefficient**	**SE**	**p**	**IR**	**CI^**§**^**
Intercept^†^	−1.71	0.06			
Bedding type
RMS	−0.07	0.16	0.65	0.93	0.68, 1.28
Straw	Ref				
Housing type^ 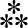 ^
Free stall	−0.04	0.17	0.82	0.96	0.69, 1.34
Tie stall	Ref				
Bedding depth^ 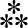 ^
≥10 cm	0.09	0.19	0.65	1.09	0.75, 1.59
<10 cm	Ref				
Stall age^‡^, ^ 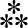 ^	0.07	0.04	0.08	1.07	0.99, 1.19
Herd size^ 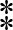 , 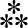 ^	−0.14	0.11	0.22	0.87	0.70, 1.08
Herd size^2^	8.20E-6	0.00	<0.01		
Herd size^3^	−8.26E-9	0.00	<0.01		
Variance
Farm	0.10				

§ *Confidence interval of the incidence ratio (IR)*.

† *Stall age and herd size were centered on 5 years and 100 cows, respectively. The intercept, therefore, represents the cow's log risk of having a linear score >4.0 for a cow in a 100 milking cow herd that had renovated its stalls 5 years ago*.

‡ *Coefficient represent an increase of 10 years*.

^
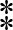
^*Coefficient represent an increase of 100 cows*.

^
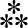
^*Putative confounders*.

### Model 3: Impact of Bedding Type on Risk of Acquisition of SCM

**Figure 4:**
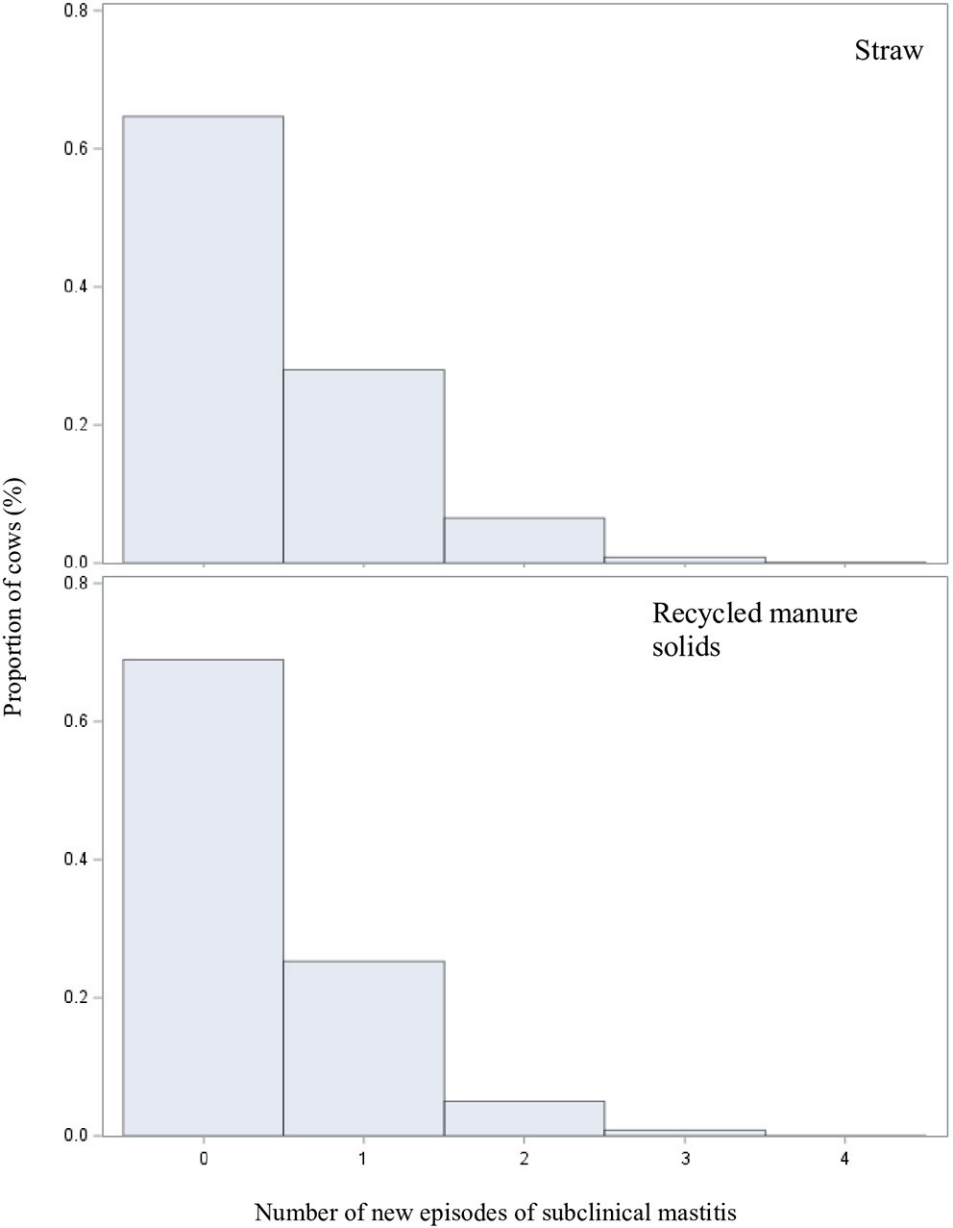
Distributions of number of newly acquired subclinical mastitis (defined as a linear score ≥ 4.0 on first test followed by a linear score =4.0 on second test) for a given cow by bedding type in 43,546 pairs of DHI tests from 11,031 cows from 60 straw-bedded farms (top) and 20 recycled manure solids farms (bottom).

In this last model we used 43,546 pairs of DHI tests from 11,031 cows. Distribution of incidence of SCM episodes by bedding type is illustrated in Figure 4. Again, the relationship between herd size and the outcome appeared to be non-linear on the logarithmic scale. The square and cubic polynomial herd size terms were, therefore, retained in the model. After controlling the putative confounders, the risk of acquiring a new SCM in RMS-bedded cows was estimated to be 0.73 times (%95 CI: 0.54, 1.00) that of cows housed on straw (Table 4).

**Table 4:** Risk of acquiring a new subclinical mastitis as function of bedding type estimated using a generalized linear mixed model applied to 43,546 pairs of DHI tests from 11,031 cows from 20 RMS farms and 60 straw-bedded farms.

	**Coefficient**	**SE**	**p**	**IR**	**CI^**§**^**
Intercept^†^	−5.88	0.06			
Bedding type
RMS	−0.31	0.16	0.05	0.73	0.54, 1.00
Straw	Ref				
Housing type^ 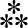 ^
Free stall	0.22	0.18	0.24	1.24	0.88, 1.77
Tie stall	Ref				
Bedding depth^ 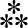 ^
≥10 cm	0.17	0.18	0.36	1.19	0.83, 1.69
<10 cm	Ref				
Stall age^‡^, ^ 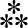 ^	0.05	0.04	0.16	1.05	0.97, 1.14
Herd size^ 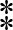 , 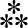 ^	0.05	0.10	0.64	1.05	0.86, 1.28
Herd size^2^	−0.10E-4	0.00	<0.01		
Herd size^3^	1.58E-8	0.00	<0.01		
*Variance*
Farm	0.08				

§ *Confidence interval of the incidence ratio (IR)*.

† *Stall age and herd size were centered on 5 years and 100 cows, respectively. The intercept, therefore, represents the cow's log risk of acquiring a new subclinical mastitis for a cow in a 100 milking cows herd that had renovated its stalls 5 years ago*.

‡ *Coefficient represent an increase of 10 years*.

^
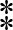
^*Coefficient represent an increase of 100 cows*.

^
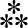
^*Putative confounders*.

## Discussion

We analyzed SCM dynamics, in small to medium size herds typical of Eastern Canada, using three different measures and we were not able to find statistically significant associations between use of RMS and the prevalence nor incidence of SCM in any of our models. The mean LS was slightly in favor of straw-bedded cows. On the other hand, RMS-bedded cows were at slightly lower risk of having a LS ≥ 4.0 and of having an incident SCM. In general, we can conclude that cows on RMS farms were, at least, not at a greater risk of SCM than cows from straw-bedded farms.

We previously identified that pathogens were generally, found in greater concentrations in RMS bedding than in straw (2). These elevated counts of bacteria could increase the risk of environmental intra-mammary infections (**IMI**) (15) in RMS-bedded cows. Environmental pathogens are well-known to cause mainly clinical mastitis, which will affect few animals and for a short duration of time (16). Consequently, short duration IMIs on a few cows probably has a low impact on SCC, in general, and this may explain why we did not observe any bedding effect in our models. If RMS bedding would have increased the risk of more chronic IMI, we would then have possibly captured these differences in our models. Our results are in agreement with the work of de Haas et al. (17) who have found that high cow's SCC are associated with incidence of contagious rather than environmental pathogens. Shook et al. (16) have also demonstrated that each 1-point increase in LS was associated with a 2.3% increase in prevalence of contagious pathogens while an increase of 5.5% prevalence of environmental pathogen was required to obtain an equivalent LS increase.

Rowbotham and Ruegg (6) were also unable to detect an association between bedding type (RMS vs. sand) and SCM risk in primiparous cows housed on a research facility. In our study, we extended these conclusions to multiparous cows and to cows housed in a commercial farm context. Moreover, another of our study's strengths is the number of cows recruited and followed during the study period. This is, to our knowledge, the first study of this magnitude on the topic. Furthermore, we analyzed the SCC variations with three different methods to confirm our results.

On the other hand, since this in an observational study, our project presents some limitations. First, the herd's selection was not random and since we required straw-bedded farms to be enrolled in DHI program, they may have been more aware or concerned of their cow's mammary health than the general population of dairy farmers. If this is the case, then our estimates would be biased toward a better measured udder health in straw-bedded farms than the true udder health in that population, thus biasing the comparison with RMS farms. Since RMS farms performed in general slightly better than straw farms for most outcome studied, this would only support our conclusions that RMS farms do not have worse SCM performances than straw-bedded herds. Nevertheless, our estimates of SCM incidence were very close to significant and in favor of RMS herds, and such a bias, if present, could have led to a type II error (i.e., not being able to conclude on a difference in favor of RMS farms while a true difference exist). On the other hand, a large proportion of the RMS herds recruited in our study were indeed enrolled in DHI program or had access the robotic milker-generated SCC data (20/27), so this bias, if present, is likely to be small.

A second limitation, is the potential for residual confounding. Given that the study design was an observational study design and that the exposure (i.e., bedding type) could not be randomly assign to farms, some important confounders could potentially distort the observed associations. We adjusted in our analyses for the most important theoretical confounders, but we cannot exclude the possibility of residual confounding by other unknown confounders. Nevertheless, given the important costs in machinery associated with producing RMS bedding, it would be impossible to randomly assign the bedding type to farms, thus precluding using an experimental study design on a large number of farms. One other study design that could be used is a before and after comparison in herds that have recently implemented RMS bedding, and, with such a design, any difference in other factors than bedding type occurring between the pre- and post-implementation periods could also biased the estimates. Moreover, it would be difficult to assemble a large number of farms transitioning to RMS bedding in a given region over a relatively short period of time. In the future, with more and more studies on this topic becoming available, meta-analyses could possibly help appraising our results in a larger context.

## Conclusion

We could not detect any difference in the mean lactational LS of cows bedded with RMS when compared to cows housed on straw. Moreover, we were not able to highlight difference in the risk for a given cow of having a DHI test with a LS ≥ 4.0 or with the risk of acquiring a SCM event. In general, the SCM situation did not appear worse in cows housed on RMS bedding as compared to cows housed on straw bedding.

## Data Availability Statement

The datasets presented in this study can be found in online repositories. The names of the repository/repositories and accession number(s) can be found at: https://doi.org/10.5683/SP3/GRPK8W.

## Ethics Statement

The animal study was reviewed and approved by Animal Care and Use Committee of the Faculty of Veterinary Medicine (Université de Montréal; protocol 17-Rech-1886). Written informed consent was obtained from the owners for the participation of their animals in this study.

## Author Contributions

GF, CC, and SD contributed to conception and design of the study. AF collected and analyzed the data (with help from SD) and wrote the first draft of the paper. All authors contributed to manuscript revision, approved and read the submitted version.

## Funding

This project was funded by grants from Novalait, the Consortium de recherche et innovations en bioprocédés industriels au Québec (#2015-044-C17), the Fonds Québécois de la recherche sur la nature et les technologies (#2017-LG-201835), and the Natural sciences and engineering research council of Canada (#CRDPJ 499421-2016). AF received funding and support from the Natural sciences and engineering research council of Canada Collaborative research and training experience program in milk quality, from the Canadian Dairy Commission, from Agria, and from Op+lait.

## Conflict of Interest

The authors declare that the research was conducted in the absence of any commercial or financial relationships that could be construed as a potential conflict of interest.

## Publisher's Note

All claims expressed in this article are solely those of the authors and do not necessarily represent those of their affiliated organizations, or those of the publisher, the editors and the reviewers. Any product that may be evaluated in this article, or claim that may be made by its manufacturer, is not guaranteed or endorsed by the publisher.
