# Corrigendum: Association Between Recycled Manure Solids Bedding and Subclinical Mastitis Incidence: A Canadian Cohort Study

**DOI:** 10.3389/fvets.2022.939744

**Published:** 2022-05-27

**Authors:** Annie Fréchette, Gilles Fecteau, Caroline Côté, Simon Dufour

**Affiliations:** ^1^Regroupement FRQ-NT Op+Lait, Saint-Hyacinthe, QC, Canada; ^2^Mastitis Network, Saint-Hyacinthe, QC, Canada; ^3^Department of Pathology and Microbiology, Faculty of Veterinary Medicine, Université de Montréal, Montreal, QC, Canada; ^4^Department of Clinical Sciences, Faculty of Veterinary Medicine, Université de Montréal, Montreal, QC, Canada; ^5^Research and Development Institute for the Agri-Environment (IRDA), Québec City, QC, Canada

**Keywords:** bedding, subclinical mastitis, somatic cell count, recycled manure solids (RMS), dairy cows

In the original article, there were mistakes in Tables 2, 3, and 4 as published. Data alignment problems were present in these tables. The corrected Tables 2, 3, and 4 appears below.

**Table 2:** Impact of bedding on the cow's mean lactation linear score estimated using a generalized linear mixed model using the data from 15,161 lactations of 11,031 cows from 20 recycled manure solids (RMS) farms and 60 straw-bedded farms.

	**Coefficient**	**SE**	**CI**	***p***
Intercept^†^	2.37	0.06	2.25, 2.49	
Bedding type
RMS	0.10	0.15	−0.20, 0.40	0.50
Straw	Ref			
Housing type^‡^
Free stall	0.25	0.17	−0.09, 0.59	0.15
Tie stall	Ref			
Bedding depth^ 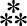 ^
≥10 cm	−0.06	0.19	−0.44, 0.32	0.77
<10 cm	Ref			
Stall age^‡, 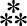 ^	0.09	0.04	0.01, 0.17	0.04
Herd size^ 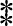 , 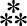 ^	−0.11	0.04	−0.2, −0.02	0.01
Variance
Farm	0.11			
Cow	0.93			
Lactation	1.88			

^†^*Stall age and herd size were centered on 5 years and 100 cows, respectively. The intercept, therefore, represents the cows' mean LS for a cow in a 100 milking cows herd that had renovated its stalls 5 years ago*.

^‡^*Coefficient represent an increase of 10 years*.

^
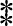
^*Coefficient represent an increase of 100 cows*.

^
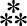
^*Putative confounders*.

**Table 3:** Impact of bedding type on the risk of a DHI test with a linear score >4.0 in 11,031 cows from 20 RMS farms and 60 straw-bedded farms and estimated using a generalized linear mixed model.

	**Coefficient**	**SE**	**p**	**IR**	**CI^**§**^**
Intercept^†^	−1.71	0.06			
Bedding type
RMS	−0.07	0.16	0.65	0.93	0.68, 1.28
Straw	Ref				
Housing type^ 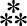 ^
Free stall	−0.04	0.17	0.82	0.96	0.69, 1.34
Tie stall	Ref				
Bedding depth^ 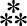 ^
≥10 cm	0.09	0.19	0.65	1.09	0.75, 1.59
<10 cm	Ref				
Stall age^‡^, ^ 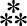 ^	0.07	0.04	0.08	1.07	0.99, 1.19
Herd size^ 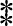 , 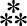 ^	−0.14	0.11	0.22	0.87	0.70, 1.08
Herd size^2^	8.20E-6	0.00	<0.01		
Herd size^3^	−8.26E-9	0.00	<0.01		
Variance
Farm	0.10				

§ *Confidence interval of the incidence ratio (IR)*.

† *Stall age and herd size were centered on 5 years and 100 cows, respectively. The intercept, therefore, represents the cow's log risk of having a linear score >4.0 for a cow in a 100 milking cow herd that had renovated its stalls 5 years ago*.

‡ *Coefficient represent an increase of 10 years*.

^
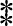
^*Coefficient represent an increase of 100 cows*.

^
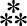
^*Putative confounders*.

**Table 4:** Risk of acquiring a new subclinical mastitis as function of bedding type estimated using a generalized linear mixed model applied to 43,546 pairs of DHI tests from 11,031 cows from 20 RMS farms and 60 straw-bedded farms.

	**Coefficient**	**SE**	**p**	**IR**	**CI^**§**^**
Intercept^†^	−5.88	0.06			
Bedding type
RMS	−0.31	0.16	0.05	0.73	0.54, 1.00
Straw	Ref				
Housing type^ 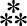 ^
Free stall	0.22	0.18	0.24	1.24	0.88, 1.77
Tie stall	Ref				
Bedding depth^ 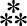 ^
≥10 cm	0.17	0.18	0.36	1.19	0.83, 1.69
<10 cm	Ref				
Stall age^‡^, ^ 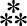 ^	0.05	0.04	0.16	1.05	0.97, 1.14
Herd size^ 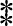 , 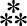 ^	0.05	0.10	0.64	1.05	0.86, 1.28
Herd size^2^	−0.10E-4	0.00	<0.01		
Herd size^3^	1.58E-8	0.00	<0.01		
*Variance*
Farm	0.08				

§ *Confidence interval of the incidence ratio (IR)*.

† *Stall age and herd size were centered on 5 years and 100 cows, respectively. The intercept, therefore, represents the cow's log risk of acquiring a new subclinical mastitis for a cow in a 100 milking cows herd that had renovated its stalls 5 years ago*.

‡ *Coefficient represent an increase of 10 years*.

^
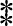
^*Coefficient represent an increase of 100 cows*.

^
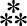
^*Putative confounders*.

The authors apologize for these errors and state that this does not change the scientific conclusions of the article in any way. The original article has been updated.

## Publisher's Note

All claims expressed in this article are solely those of the authors and do not necessarily represent those of their affiliated organizations, or those of the publisher, the editors and the reviewers. Any product that may be evaluated in this article, or claim that may be made by its manufacturer, is not guaranteed or endorsed by the publisher.

